# Enhancing cardiovascular patients’ knowledge of air pollution: a pilot study evaluating the impact of an educational intervention in cardiac rehabilitation

**DOI:** 10.3389/fresc.2024.1495621

**Published:** 2024-11-22

**Authors:** Marta Supervia, Ana Paula Delgado Bomtempo, Eduardo Gómez, Amanda R. Bonikowske, Maria Olga Arroyo-Riaño, Gabriela Lima de Melo Ghisi

**Affiliations:** ^1^ Department of Physical Medicine and Rehabilitation Gregorio Marañón General University Hospital, Gregorio Marañón Health Research Institute, Madrid, Spain; ^2^ Radiology, Rehabilitation and Physiotherapy Department, Complutense University School of Medicine, Madrid, Spain; ^3^ Division of Preventive Cardiology, Department of Cardiovascular Medicine, Mayo Clinic, Rochester, NY, United States; ^4^ Graduate Program in Physical Education, Faculty of Physical Education and Sports, Federal University of Juiz de Fora, Juiz de Fora, Brazil; ^5^ KITE Research Institute, University Health Network, Toronto, ON, Canada; ^6^ Department of Physical Therapy, University of Toronto, Toronto, ON, Canada

**Keywords:** air pollution, cardiovascular health, patient education, cardiac rehabilitation, environmental pollution

## Abstract

**Introduction:**

Air pollution poses significant risks to cardiovascular health, yet patients often lack comprehensive knowledge about its impact and mitigation strategies. This study aims to evaluate the effectiveness of an educational intervention within a cardiac rehabilitation (CR) context in enhancing patients’ understanding of air pollution and its cardiovascular effects.

**Methods:**

A pre-post pilot study was conducted from February 2021 to June 2021 at the Gregorio Marañón University General Hospital, Madrid, Spain. A total of 43 patients with cardiovascular disease attending CR were enrolled. Participants received a 1-h educational intervention delivered either in-person or online, focusing on the effects of air pollution and strategies to reduce exposure. Pre- and post-intervention questionnaires assessed participants’ knowledge and perceptions. Descriptive statistics were used to analyze changes in awareness and understanding.

**Results:**

Initially, participants demonstrated a high level of awareness about the health impacts of air pollution, with 100% acknowledging its effects on health. However, detailed knowledge about specific concepts such as the Air Quality Index (AQI) and particulate matter (PM2.5) was limited. Post-intervention, there was a significant increase in knowledge, with familiarity with AQI rising from 61% to 81% (*p* = 0.02) and understanding of PM2.5 improving from 28% to 58% (*p* = 0.01). This indicates that the educational intervention effectively bridged gaps in understanding and reinforced the importance of environmental factors in cardiovascular health management.

**Discussion:**

The pilot study highlights the critical role of targeted education in improving patient awareness and knowledge about air pollution. The significant improvement in understanding key concepts underscores the need for broader educational initiatives that extend beyond CR programs. Future research should explore the impact of such interventions on long-term health outcomes and consider expanding educational efforts to include healthcare providers and family members.

## 1 Introduction

Cardiovascular disease (CVD) remains the leading cause of morbidity and mortality worldwide, significantly burdening health systems and economies (1–3). This global health crisis is driven by several well-known risk factors, including hypertension, high cholesterol, smoking, diabetes, obesity, and physical inactivity (4). The causality of these “classic” risk factors is well understood, and they are widely used to evaluate overall cardiovascular risk in the general population (4, 5). However, in the last decades, emerging risk factors have gained recognition for their significant contribution to the development and progression of cardiovascular disease (6, 7). Among them, air pollution has become a growing concern due to its substantial and increasing impact on cardiovascular health (8).

Air pollution is a significant global health threat, responsible for an estimated 12% of all deaths in 2019 (9). Remarkably, it accounts for nearly 20% of cardiovascular disease deaths (8, 10), positioning it as one of the leading modifiable risk factors for mortality – surpassing even high LDL cholesterol, physical inactivity, high body-mass index, and alcohol use (8). The complexity of air pollution - comprising particulate matter (PM), ozone (O_3_), and nitrogen dioxide (NO_2_) - and its widespread, fluctuating presence underscores its pervasive health impacts (11). These pollutants originate from various sources, undergo atmospheric transformations, and affect populations across time and space, creating unique challenges for public health and regulatory efforts (11, 12). Certain groups, including the elderly, socio-economically disadvantaged, and those with pre-existing conditions like obesity or diabetes, are particularly susceptible, while vulnerable populations are those exposed to higher pollution levels due to factors such as proximity to industrial emissions, heavy traffic, or wildfires (13–15).

Cardiac rehabilitation (CR) is a Class 1A recommendation for individuals living with CVD (16, 17). It is a multifaceted program involving medical assessment, structured exercise training, lifestyle counseling, cardiovascular risk factor management, psychosocial support and patient education (18, 19), all aimed at aiding recovery, improving physical fitness, and enhancing overall quality of life (20). CR offers a crucial opportunity to address the impact of air pollution on cardiovascular health by integrating education on reducing exposure, thereby empowering patients to mitigate their cardiovascular risks and advocate for policies that promote cleaner air (21, 22).

Despite the well-established link between air pollution and cardiovascular risk, awareness of this connection remains limited among both patients and the broader community (23–26). Most efforts in CVD management focus on traditional risk factors, with little emphasis placed on environmental risks like air pollution (10). Furthermore, there is a lack of studies examining the awareness and knowledge of CR participants regarding the impact of air pollution on cardiovascular health. This pilot study aims to fill this gap by exploring the level of knowledge and perceptions among CR participants and assessing whether a brief educational intervention can effectively raise awareness and improve understanding of the cardiovascular risks associated with air pollution.

## 2 Materials and methods

### 2.1 Design and procedures

A pre-post design was used for this pilot study, which was conducted from February 2021 to June 2021. Patients living with CVD and attending a CR program received the brief education intervention and completed pre- and post-intervention questionnaires. The study was conducted in the Gregorio Marañón University General Hospital (Madrid, Spain) following the standards required by the Declaration of Helsinki and approved by the Hospital's Ethics Committee (RCAcont, 17032021). All participants provided written informed consent.

Eligible patients were approached upon their arrival for the baseline CR assessment. After obtaining consent, participants were scheduled for an educational session and given the option to choose between attending in-person or participating virtually. Participants completed the pre-questionnaire prior to the educational session. For those in the face-to-face format, the follow-up survey was administered immediately after the session concluded. Participants in the online format were asked to complete the follow-up survey during their next visit to the rehab center. Only the data from participants who completed both the pre- and post-questionnaires were included in the analysis.

### 2.2 Educational intervention

The educational intervention consisted of a 1-h lecture delivered by the CR healthcare team, enhanced with a PowerPoint presentation and accompanied by an informative leaflet summarizing the key messages. The lecture focused on practical recommendations for reducing exposure to air pollution and understanding its associated risks. Specific educational topics included strategies for minimizing exposure, identifying common sources of pollution, and adopting lifestyle changes to mitigate health impacts. To ensure accessibility for all patients, the intervention was available in two formats: online, for those unable to attend in person due to COVID-19 vulnerability, scheduling conflicts, or other reasons, and face-to-face, conducted during in-person CR sessions.

### 2.3 Participants

In pilot studies, power calculations are typically not required (27). However, it is generally recommended to include at least 30 subjects to estimate a parameter effectively, which is a common practice in studies involving psychosocial interventions (27, 28). In this study, we aimed for the upper limit of this range, seeking to recruit a minimum of 30 participants. Exclusion criteria encompassed individuals who lacked proficiency in Spanish or had visual or cognitive impairments that would prevent them from completing the surveys.

### 2.4 Measures

The pre- and post-intervention questionnaires were meticulously designed by the research team to evaluate participants’ knowledge and perceptions concerning environmental and atmospheric pollution, with a particular focus on its effects on cardiovascular health. These questionnaires were informed by position statements related to CVD and air pollution (8), ensuring a comprehensive assessment of the participants’ understanding and awareness of these issues.

In total, 45 questions were identical across both the pre- and post-intervention surveys. Questions were developed as yes/no, multiple choice and Likert-type scale (ranging from 0 to 5) to track changes in knowledge and attitudes over time. The primary areas of inquiry are described in Table 1. In addition, the pre-intervention questionnaire included a question assessing whether participants felt they received adequate information from healthcare professionals about atmospheric pollution following their cardiac event or CVD diagnosis. In contrast, the post-intervention questionnaire featured 8 questions focusing on the perceived usefulness and relevance of the educational content provided. The pre- and post-intervention questionnaires are available in Supplementary Appendix 1.

**Table 1:** Primary areas of inquiry included in the pre- and post-intervention questionnaires.

Section name	Number of questions	Goal of section
Environmental and atmospheric pollution knowledge	18	Assesses knowledge and understanding of environmental and atmospheric pollution, including key concepts such as the AQI, pollution sources, monitoring stations, and the impact of pollution on different environments.
Health impact of pollution (general and CVD health)	6	Assesses perceptions of how pollution affects health, focusing on cardiovascular health, the importance of avoiding polluted areas, awareness of health consequences, and the benefits of green spaces in urban environments.
Pollution patterns and preventive measures	7	Examines factors affecting atmospheric pollution levels, including the influence of time of day and vegetation in cities. It also evaluates individual actions and mitigation measures, such as the effectiveness of different types of masks in reducing exposure to airborne pollutants.
Access to information and media influence	9	Assesses your knowledge and opinions on accessing information about air pollution and the Air Quality Index, including the use of websites, mobile applications, and public resources. It also explores how media influence affects your perceptions of atmospheric pollution and its risks.
Factors influencing cardiovascular health	5	Evaluates your perceptions of the importance of various factors for cardiovascular health, including regular physical exercise, a healthy diet, avoiding tobacco exposure, following prescribed treatments, and minimizing exposure to high levels of atmospheric pollution.

AQI, air quality index; CVD, cardiovascular; NDVI, normalized difference vegetation index.

Only included at the post-intervention questionnaire.

Sociodemographic and clinical characteristics of participants were assessed through self-reported data and extracted from medical records, respectively. This included age, gender, employment status, educational level, CR referral diagnosis, usual mode of transportation and exercise habits (frequency and places where participants exercise).

### 2.5 Data analysis

SPSS Version 29.0 was used for all the analysis. All initiated surveys that had at least one response were included. The number of responses for each question varied due to missing data (e.g., respondent did not answer a question due to inapplicability, skip logic, or decided to not answer for other reasons); for descriptive analyses, percentages were computed using the number of valid responses for the specific item as the denominator. Descriptive statistics (e.g., frequency with percentage) were applied for all items in the survey. Changes in outcomes from pre- to pos*t*-test in each group were assessed using paired samples *t*-tests or chi-square analyses, as applicable.

## 3 Results

### 3.1 Participants characteristics

A convenience sample of 43 patients (mean age = 55.6 ± 11.2 years old, 12% female) agreed to participate in this study. Sociodemographic and clinical characteristics of the participants are presented in Table 2. A large portion of participants were either unemployed or on medical leave, with a significant number also being retired. In terms of education, the majority had completed high school or obtained a college/trades certificate. The most common cardiac diagnosis among participants was ischemic heart disease. Most participants used a car as their primary mode of transportation. The majority reported exercising at least twice a week, with half of the participants exercising primarily in urban environments. Additionally, 14 (33%) of participants reported that they receive adequate information from healthcare professionals regarding atmospheric pollution.

**Table 2:** Sociodemographic and clinical characteristics of participants (*N* = 43).

Characteristic	*n* (%)
Age	
Less than 56 years old	22 (51.2%)
56 years old or older	21 (48.8%)
Gender	
Male	38 (88.4%)
Female	5 (11.6%)
Employment status	
Unemployed or medical leave	18 (41.9%)
Retired	14 (32.6%)
Employed (full-time, part-time or self-employed)	10 (23.3%)
Other	1 (2.2%)
Educational level	
Elementary	7 (16.3%)
High school	13 (30.2%)
College or trades certificate	14 (32.6%)
University	9 (20.9%)
CR referral diagnosis	
Ischemic heart disease	29 (67.4%)
Bypass surgery	4 (9.3%)
Valve surgery	4 (9.3%)
Heart failure	3 (7.0%)
Congenital heart disease	1 (2.3%)
Atrial fibrillation	1 (2.3%)
Usual mode of transportation	
Car	28 (65.1%)
Subway	5 (11.6%)
Bus	4 (9.3%)
Bicycle	1 (2.3%)
Exercise habits: frequency	
Twice/week	24 (66.7%)
Sometimes	10 (27.8%)
Never	2 (5.6%)
Exercise habits: environment	
Urban area	19 (50.0%)
Green spaces	11 (28.9%)

### 3.2 Environmental and atmospheric pollution knowledge

**Figure 1:**
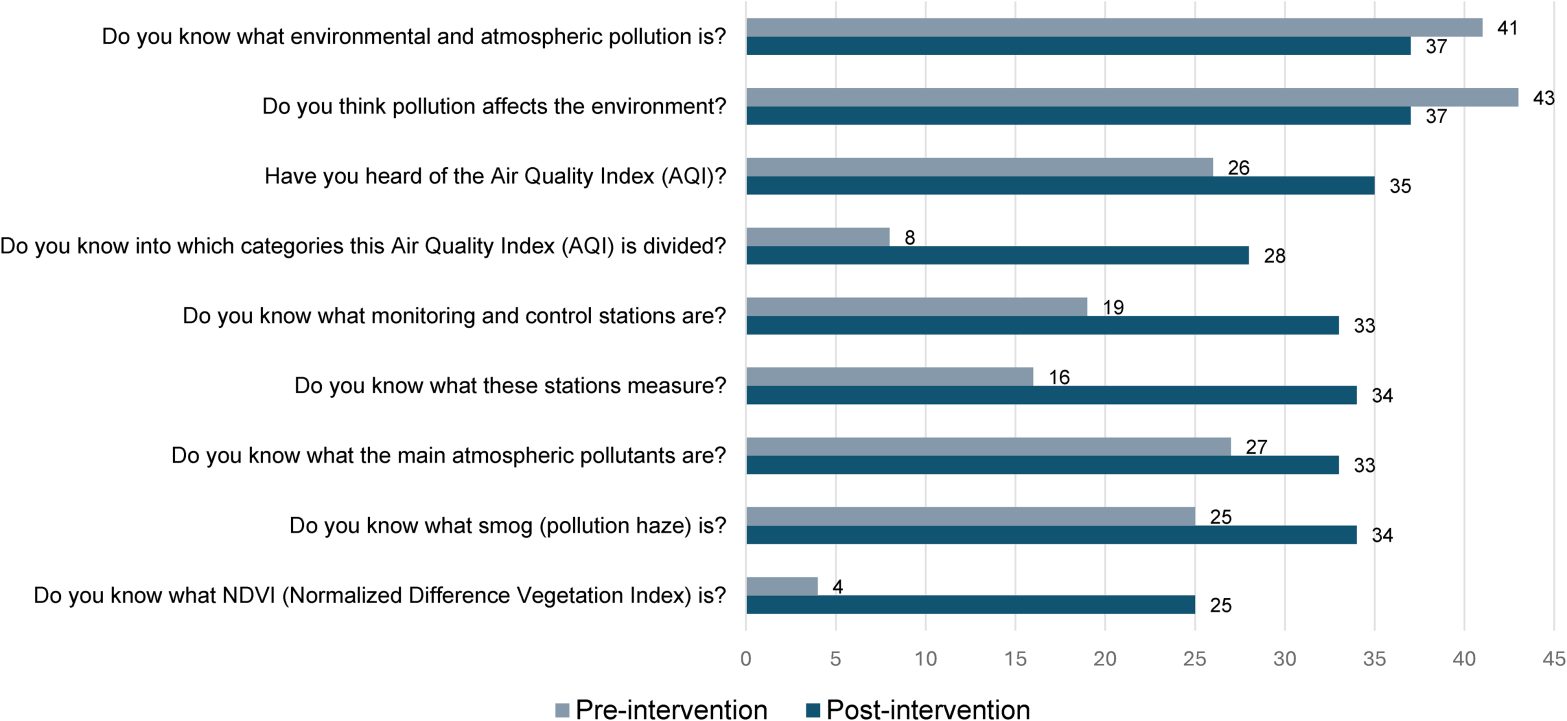
Environmental and atmospheric pollution knowledge pre- to post-intervention (*N* = 43).

Initially, 93% (*n* = 41) of participants reported they knew what environmental pollution was, and 100% (*n* = 43) recognized its impact on the environment. Following the educational intervention, there was a notable improvement in participants’ understanding of environmental and atmospheric pollution (Figure 1). Post-intervention, familiarity with the Air Quality Index (AQI) increased from 61% (*n* = 26) to 81% (*n* = 35), with 79% (*n* = 34) correctly identifying what it measures (*p* = 0.02). Awareness of AQI categories improved significantly, jumping from 19% (*n* = 8) to 65% (*n* = 28; *p* < 0.001), and knowledge of monitoring and control stations increased from 44% (*n* = 19) to 77% (*n* = 34; *p* = 0.001), with 79% (*n* = 34) understanding what these stations measure. Additionally, recognition of the primary atmospheric pollutants rose from 63% (*n* = 27) to 77% (*n* = 33) from pre- to post-intervention (*p* = 0.04).

Participants also demonstrated a better grasp of pollution sources and specific pollutants. Before the intervention, 91% (*n* = 39) of participants correctly identified anthropogenic sources as the most polluting, a percentage that slightly decreased to 84% (*n* = 36) post-intervention. Awareness of atmospheric pollutants like CO, NO_2_, and PM2.5 improved, with PM2.5 knowledge rising from 28% (*n* = 12) to 58% (*n* = 25; *p* = 0.01). Additionally, 79% (*n* = 34) of participants understood what smog is, up from 58% (*n* = 25) at pre-intervention (*p* = 0.03), and knowledge of the NDVI (Normalized Difference Vegetation Index) saw a considerable increase from 9% (*n* = 4) to 58% (*n* = 25; *p* < 0.001). Finally, understanding of the environmental impacts of agriculture and livestock improved from 49% (*n* = 21) to 74% (*n* = 32) from pre- to post-intervention (*p* = 0.02).

### 3.3 Health impact of pollution (general and CVD health)

The awareness of pollution's impact on health was high among participants at pre-intervention, with 100% acknowledging that pollution affects health. When asked to rate how much air pollution affects cardiovascular health on a scale from 0 (no impact) to 5 (a lot of impact), participants consistently rated its impact as significant both before and after the intervention [pre median = 4 (CI: 3–5) vs. post median = 5 (CI: 4–5)], reflecting heightened recognition of its serious effects. Similarly, the importance of avoiding areas with high pollution levels for cardiovascular health also scored high (i.e., 5), demonstrating strong agreement both before and after the intervention.

Participants’ knowledge of specific health effects associated with pollution increased post-intervention. Initially, all participants recognized the link between pollution and respiratory diseases, and while this number slightly decreased at post-intervention, awareness of CVD as a consequence of pollution increased from 67% (*n* = 29) to 77% (*n* = 33; *p* = 0.04). In addition, nearly all participants agreed on the beneficial health effects of green areas in cities, with 97% (*n* = 42) agreeing initially and 100% (*n* = 43) agreeing after the intervention. Most respondents, 88% (*n* = 38) pre-intervention and 91% (*n* = 39) post-intervention, indicated their preference for green areas away from cities for physical exercise.

### 3.4 Pollution patterns and preventive measures

Pollution patterns and preventive measures provide important insights into patients’ understanding of air quality dynamics. The results indicate that there was no notable change in perceptions before and after the intervention. Most respondents incorrectly believed that pollution levels are lowest in the morning and that atmospheric pollution decreases during cloudy or sunny days. Notably, no respondents correctly identified rainy weather or windy conditions as factors that reduce pollution levels.

Regarding urban vegetation, there is a strong consensus (95%, *n* = 41) that vegetation in cities contributes positively to reducing pollution, with no notable differences in preferences for vegetation management before and after the educational intervention. While some respondents advocate for a diverse and widespread array of plants (32%, *n* = 14), others emphasize the importance of controlling species and placement (35%, *n* = 15), reflecting a balanced view on how urban greenery should be managed for optimal health benefits.

Individual actions to mitigate pollution are also notable. Reducing plastic consumption (93%, *n* = 40) and cutting down on water and electricity usage (65%, *n* = 28) are widely recognized as effective measures. Additionally, the preference for wearing filtering masks, particularly N95 masks, increased significantly after the educational intervention, rising from 65% (*n* = 28) to 79% (*n* = 34; *p* = 0.04). Meanwhile, the preference for other types of masks, such as disposable surgical masks, decreased significantly from 32% (*n* = 14) to 12% (*n* = 5; *p* = 0.02). This shift underscores a growing awareness of the superior effectiveness of N95 masks in protecting against airborne pollutants.

### 3.5 Access to information and Media influence

The results reveal a substantial improvement in awareness and knowledge about air quality information following the intervention. Initially, only 19% (*n* = 8) of respondents were aware of online portals or websites to check air pollution and the AQI, but this figure increased to 60% (*n* = 26) at post-intervention (*p* < 0.001). Similarly, awareness of how to access this information through public institutions rose from 22% (*n* = 9) to 73% (*n* = 27; *p* < 0.001), and knowledge of mobile applications for monitoring air quality improved from 11% (*n* = 5) to 52% (*n* = 19; *p* = 0.002).

Respondents generally rated the importance of receiving information about atmospheric pollution and available resources highly, and similarly rated the potential benefits of this information for cardiovascular health. A majority of patients (61%, *n* = 26) felt that media such as television, radio, and social networks have influenced their opinions on atmospheric pollution. However, opinions on whether this media exposure has led to an overestimation or underestimation of pollution risks varied: 42% (*n* = 11) believed that media coverage has led to an overestimation of risks, 27% (*n* = 7) felt it has led to an underestimation, and 32% (*n* = 9) considered the media's influence to be neutral or not impactful.

### 3.6 Factors influencing cardiovascular health

**Figure 2:**
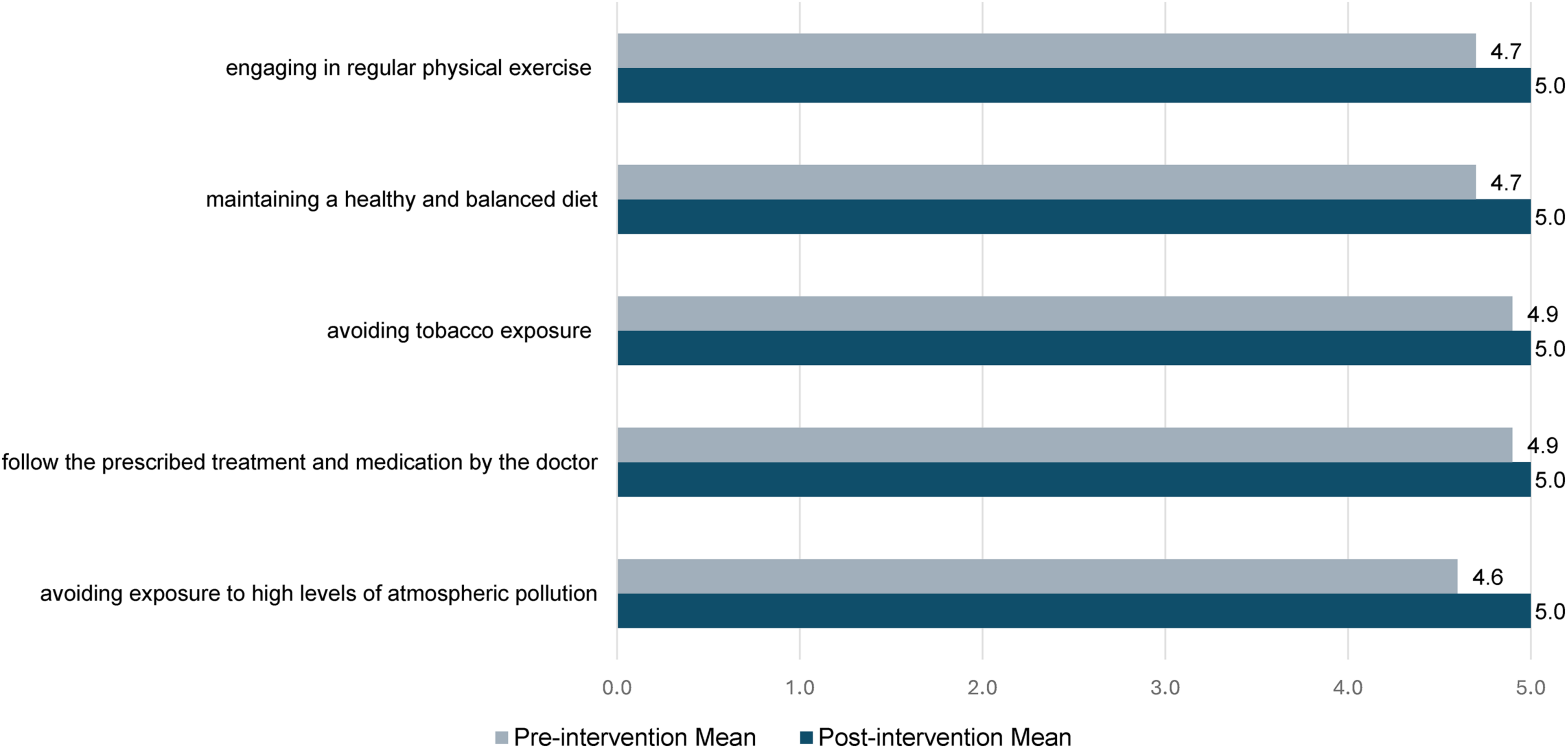
Responses to Likert-type questions (0 = not at all, 5 = very important) on the importance of factors for cardiovascular health (*N* = 43), means are reported.

Figure 2 illustrates the responses to Likert-type questions, where participants rated the importance of various factors for cardiovascular health from 0 (not at all) to 5 (a lot). Post-intervention, there was a marked increase in the perceived importance of all factors compared to pre-intervention ratings, which were already high. Notably, the perception of avoiding exposure to high levels of atmospheric pollution saw the most significant rise, with the mean score increasing from 4.6 ± 0.7 to 5.0 ± 0.4 (*p* = 0.01).

### 3.7 Perceived usefulness and relevance of the educational intervention provided

The data on the perceived usefulness and relevance of the educational intervention reveals strong participant engagement. Out of 43 respondents, 98% (*n* = 39) indicated they will consider the information about air pollution in their daily lives. Additionally, 87% (*n* = 32) plan to consult air pollution levels more frequently, and 81% (*n* = 29) intend to download the mobile applications recommended during the sessions. Participants rated the usefulness of the information about atmospheric pollution, air pollutants, and the AQI very highly, with scores ranging from 4 to 5 out of 5. The educational session was also deemed entertaining, with similar high ratings. Furthermore, 92% (*n* = 31) agreed that atmospheric pollution should be included in educational sessions of the CR Program.

## 4 Discussion

This pilot study aimed to evaluate the impact of an educational intervention on CR patients’ knowledge and perceptions regarding atmospheric pollution and its effects on cardiovascular health. To our knowledge, this is the first study to integrate educational content on atmospheric pollution specifically within a CR context, recognizing this setting a prime opportunity to connect with individuals who are at increased risk of adverse health outcomes from such exposure (21, 22). Key findings from the intervention include a significant improvement in participants’ understanding of air pollution, heightened awareness of the AQI, and an increased perception of the importance of avoiding high pollution areas for cardiovascular health. Participants also demonstrated high satisfaction with the educational sessions, rating the information as highly useful and relevant. The intervention positively influenced participants’ knowledge of effective protective measures and resources for monitoring air quality, reflecting their strong engagement and appreciation of the content provided.

Air pollution has well-documented detrimental effects on cardiovascular health (11, 29), making it crucial for patients to understand its impact and take preventive measures. Educating patients about the links between air pollution and CVD is essential for empowering them to make informed decisions about their health (30). For instance, understanding the AQI allows patients to decide whether it is safe to exercise outdoors based on current air quality conditions (31, 32). Knowing about specific pollutants, such as PM2.5, helps individuals recognize the types of pollutants they should avoid and the importance of using protective measures, like N95 masks, on days with high pollution levels (33). The results from this study highlight that although patients had a strong awareness of the health impacts of pollution - 100% acknowledging its effect on health - not all key concepts were known. Following the educational intervention, there was a significant increase in understanding of the AQI and specific atmospheric pollutants like PM2.5, among other important topics. This brief educational intervention effectively bridged gaps in awareness and reinforced the role of environmental factors in managing cardiovascular health.

The pilot study findings highlight a complex relationship between media influence and patient behavior regarding air pollution. Although a significant majority of participants acknowledged that media such as television, radio, and social networks influenced their views on atmospheric pollution, there was considerable variation in perceptions of this influence. Some participants felt that media coverage exaggerated pollution risks, potentially leading to heightened anxiety or misinformed protective behaviors (34, 35), while others thought media coverage downplayed the risks, potentially leading to complacency (35). This divergence emphasizes the critical role of education in equipping patients with accurate, actionable information (36, 37). Educational interventions can counteract the potential for misleading media narratives (38, 39) by providing clear, evidence-based guidance on understanding and managing air quality. For instance, by improving awareness of reliable sources for air quality data and explaining how to interpret the AQI, educational programs can help patients discern between sensationalized media reports and scientifically grounded information. Furthermore, educating patients about the specific health impacts of pollutants and the benefits of practical measures, such as monitoring air quality before outdoor exercise, could mitigate the effects of media misinformation. By reinforcing accurate knowledge and directing patients to credible resources, formal and reliable education empowers individuals to make informed decisions and adopt behaviors that effectively manage their exposure to environmental pollutants, ultimately supporting better cardiovascular health outcomes.

The results from this pilot study have implications for both practice and future research. Integrating educational content on air pollution into CR programs, as demonstrated here, can enhance patients’ understanding of environmental factors impacting cardiovascular health. This approach not only bridges knowledge gaps but could also empower patients to make informed decisions about their health, such as using the AQI to determine safe times for outdoor exercise and recognizing the importance of filtering masks in mitigating exposure to pollutants. To translate these findings into practice, healthcare providers should consider incorporating environmental health education into routine care, particularly for patients with cardiovascular conditions. This could involve developing tailored educational materials, offering workshops, and utilizing digital tools to keep patients informed about air quality. Additionally, creating partnerships with public health agencies could facilitate access to reliable air quality data and resources. Future research should focus on evaluating the long-term impact of such educational interventions on patient behavior and health outcomes. Studies could explore how sustained education affects adherence to protective measures and whether improvements in environmental awareness translate into reduced cardiovascular events. Additionally, research should investigate the effectiveness of various educational strategies across different patient populations and settings. Exploring the role of media in shaping perceptions and behaviors related to air pollution can also provide insights into optimizing educational interventions and improving public health communication.

The study presents several limitations that should be considered when interpreting the results. First, the use of a pre-post design without a control group limits the ability to establish causality and control for external factors that might influence changes in knowledge and attitudes. The study's reliance on a convenience sample from a single CR program may also impact the generalizability of the findings to other settings or populations. Additionally, the sample consisted exclusively of patients with CVD in CR and had a lower proportion of females (*n* = 5), which may introduce bias. Given that only a small percentage of CVD patients attend these programs (40), this approach may exclude a significant number of individuals who could benefit from such educational interventions. It is also important to consider expanding the reach of educational efforts to include not only patients but also healthcare providers and family members or friends of these patients. Broadening the audience can enhance the overall impact of the intervention and ensure that a wider support network is equipped with the knowledge needed to manage and mitigate the effects of air pollution on cardiovascular health. The data collection process relied on self-reported measures using a questionnaire that was not psychometrically validated, making the results subject to potential bias and inaccuracies. Additionally, variability in the timing of post-intervention surveys between online and face-to-face participants may have introduced inconsistencies in assessing immediate and retained knowledge. For the online group, the post-survey was conducted after a week, raising the possibility that participants may have acquired knowledge through self-education or other means. Furthermore, evaluating actual behavior changes and long-term knowledge retention would require a longer follow-up period. Future research should address these limitations by incorporating control groups, using larger and more diverse samples with validated tools, and extending follow-up periods to evaluate sustained effects and behavioral changes.

In conclusion, this pilot study describes our initial efforts to integrate environmental pollution education into CR, showing significant improvements in patients’ understanding of key concepts like the AQI. By empowering participants with this knowledge, we could better equip them to manage their cardiovascular health in the context of environmental risks. The findings underscore the need for broader educational initiatives, which could enhance patient awareness and may also drive meaningful improvements in public health outcomes.

## Data Availability

The raw data supporting the conclusions of this article will be made available by the authors, without undue reservation.
