# Supplementary material for: Enhancing cardiovascular patients’ knowledge of air pollution: a pilot study evaluating the impact of an educational intervention in cardiac rehabilitation
Source: Front Rehabil Sci. 2024 Nov 22;5:1495621. doi: 10.3389/fresc.2024.1495621 (PMC11621042; doi:10.3389/fresc.2024.1495621)
Supplement: Supplementary file 1 [file Supplementaryfile1.docx]

Appendix 1: Detailed questionnaire structure and response options

| **Section** | **Question** | **Response Options** |
| --- | --- | --- |
| Environmental and Atmospheric Pollution Knowledge | Do you know what environmental and atmospheric pollution is? | Yes/No |
|  | Do you think pollution affects the environment | Yes/No |
|  | Have you heard of the Air Quality Index (AQI)? | Yes/No |
|  | If you answered yes to question 5, please choose what the Air Quality Index (AQI) consists of: | Choose one: The AQI is an index for reporting annual air quality. It indicates the level of atmospheric purity or pollution and the related health effects that may concern you/The AQI is an index for reporting daily air quality. It indicates the level of atmospheric purity or pollution and the related health effects that may concern you*/The AQI is an index that does not measure ground-level ozone, particulate matter pollution, carbon monoxide, sulfur dioxide, or nitrogen dioxide/The AQI is an index for reporting daily air quality. It indicates atmospheric temperature and the related health effects that may concern you. |
|  | Do you know into which categories this Air Quality Index (AQI) is divided? | Yes/No |
|  | Do you know what monitoring and control stations are? | Yes/No |
|  | Do you know what these stations measure? | Yes/No |
|  | Do you know what the main atmospheric pollutants are? | Yes/No |
|  | What type of source do you think is most polluting? | Choose one: Natural sources / Artificial or anthropogenic sources* |
|  | What types of pollution do you think exist? | Select as appropriate: Air pollution / Soil pollution / Water pollution / Park pollution / Vegetation pollution |
|  | Mark the atmospheric pollutants you know. | Select as appropriate: Ozone (O_3_) / Carbon monoxide (CO) / Nitrogen dioxide (NO_2_) / Sulfur dioxide (SO_2_) / Particulate matter PM10 / Particulate matter PM2.5 / Ultrafine particles UFP |
|  | Where does more Particulate Matter (PM10 and PM2.5) accumulate? | Choose one: High-traffic areas* / Parks and green spaces / None |
|  | What urban areas have lower levels of pollution? (Choose one) | Choose one: High-traffic areas / Parks and green spaces* / None |
|  | What is the non-natural or anthropogenic source of CO (carbon monoxide)? | Select as appropriate: Tobacco burning / Livestock fluctuations / Fuel burning / None of the above |
|  | Do you know what smog (pollution haze) is? | Yes/No |
|  | Do you know what NDVI (Normalized Difference Vegetation Index) is? | Yes/No |
|  | What uses do you think NDVI has? | Select as appropriate: Estimate the quantity, quality, and development of vegetation / Estimate the different existing plant species / Reflect land use and demographic distribution |
|  | How do you think agriculture and livestock impact environmental pollution? | Choose one: Beneficial/Harmful*/Has no effect |
| Health impact of pollution (general and CVD health) | Do you think pollution affects our health? | Yes/No |
|  | To what degree do you think air pollution affects your cardiovascular health? | Rate from 0 (nothing) to 5 (a lot) |
|  | Do you think it's important to avoid outdoor areas with high levels of pollution for your cardiovascular health? | Rate from 0 (nothing) to 5 (a lot) |
|  | Are you aware of the effects and consequences that pollution can have on your health? | Select as appropriate: Respiratory diseases/CVD/ Joint damage/Schizophrenia |
|  | Do you think the existence of green areas in cities has beneficial effects on health? | Yes/No |
|  | Which environment do you consider most appropriate and clean for physical exercise? | Choose one: Green areas away from cities*/ Green areas within cities/Urban areas/It doesn’t matter |
| Pollution Patterns and Preventive Measures | When does the level of atmospheric pollution decrease, and the number of pollutants in the air decrease? | Choose one: When it's windy*/ When it has rained* / When it's cloudy / When it's clear and sunny |
|  | At what time of day is the level of atmospheric pollution usually lower? | Choose one: In the morning / At noon / In the afternoon* / At night / Never |
|  | Do you think the existence of vegetation in cities contributes to reducing pollution? | Yes/No |
|  | Regarding the presence of vegetation in cities, what do you consider more beneficial for health and reducing pollution? | Select as appropriate: Lots of vegetation, with many different species, as widespread as possible/ Control of species, quantity, and location of vegetation/Presence of large trees on streets/Quantity and location are irrelevant, the more vegetation, the better |
|  | What are the individual actions you think can reduce environmental pollution: | Select the correct ones: Reduce water and electricity usage*/Consume genetically modified products/Consume products from large chains and multinational companies/Reduce plastic consumption*/Use private transportation |
|  | Do you think wearing a filtering mask can reduce our exposure to airborne pollutants? | Yes/No |
|  | Which mask do you think is most effective for this purpose? | Choose one: Cloth masks / Disposable surgical mask / N95 mask* |
| Access to Information and Media Influence | Do you know any portal or website to check the air pollution situation and the Air Quality Index in your city? | Yes/No |
|  | Do you know how to consult the information about the Air Quality Index and air pollution provided by public institutions in your city? | Yes/No |
|  | Do you know any mobile applications to check the air pollution situation and the Air Quality Index in your city? | Yes/No |
|  | How would you rate receiving information about atmospheric pollution, pollutants, and the Air Quality Index? | Rate from 0 (very little, not at all) to 5 (a lot) |
|  | How would you rate receiving information about available resources (websites/mobile apps) for checking pollution levels and the Air Quality Index? | Rate from 0 (not at all) to 5 (a lot) |
|  | To what extent do you think receiving this information could benefit your cardiovascular health? | Rate from 0 (not at all) to 5 (a lot) |
|  | Do you think media such as television, radio, social networks, etc., have influenced your opinion regarding atmospheric pollution? | Yes/No |
|  | How much influence do you think media such as television, radio, social networks, etc., have on your opinion regarding atmospheric pollution? | Rate from 0 (very little, not at all) to 5 (a lot) |
|  | Your opinion on the risks posed by high levels of atmospheric pollution, influenced by the aforementioned media, tends to: | Select as appropriate: Underestimate/Overestimate /It doesn’t matter |
| Factors Influencing Cardiovascular Health | **How important do you think regular physical exercise is for your cardiovascular health?** | Rate from 0 (not at all) to 5 (very important) |
|  | **How important do you think maintaining a healthy and balanced diet is for your cardiovascular health?** | Rate from 0 (not at all) to 5 (very important) |
|  | **How important do you think avoiding tobacco exposure is for your cardiovascular health?** | Rate from 0 (not at all) to 5 (very important) |
|  | **How important do you think it is to follow the prescribed treatment and medication by the doctor for your cardiovascular health?** | Rate from 0 (not at all) to 5 (very important) |
|  | **How important do you think avoiding exposure to high levels of atmospheric pollution is for your cardiovascular health?** | Rate from 0 (not at all) to 5 (very important) |
| Previous information received | Since your cardiac event or CVD diagnosis, do you think you receive adequate information from healthcare professionals regarding atmospheric pollution? ^a^ | Yes/No |
| Perceived Usefulness and Relevance of the Educational Intervention Provided | How do you rate the usefulness of the information received in the educational session about atmospheric pollution, air pollutants, and the Air Quality Index? ^b^ | Rate from 0 (very little, not at all) to 5 (a lot) |
|  | To what extent do you think it is important to receive information about atmospheric pollution in educational sessions regarding your cardiovascular health? ^b^ | Rate from 0 (very little, not at all) to 5 (a lot) |
|  | Do you think that atmospheric pollution should be routinely included in the information provided in educational sessions of the Cardiac Rehabilitation Program? ^b^ | Rate from 0 (very little, not at all) to 5 (very often) |
|  | How do you rate the usefulness of the information received in the educational session about available resources (websites, mobile apps, etc.) for checking atmospheric pollution levels and the Air Quality Index (AQI)? ^b^ | Rate from 0 (very little, not at all) to 5 (a lot) |
|  | Will you consider the information provided about air pollution in your daily life? ^b^ | Yes/No |
|  | Do you intend to consult air pollution levels more frequently from now on? ^b^ | Yes/No |
|  | Will you download any of the mobile applications proposed in the educational sessions for consultation? ^b^ | Yes/No |
|  | Do you think that atmospheric pollution should be routinely included in the information provided in educational sessions of the Cardiac Rehabilitation Program? ^b^ | Yes/No |

* correct answer

^a^ Only available in the pre-intervention questionnaire

^b^ Only available in the post-intervention questionnaire
